# Promising Genomic Testing for Biliary Tract Cancer Using Endoscopic Ultrasound-Guided Fine-Needle Aspiration/Biopsy Specimens

**DOI:** 10.3390/diagnostics12040900

**Published:** 2022-04-05

**Authors:** Masaki Kuwatani, Kazumichi Kawakubo, Naoya Sakamoto

**Affiliations:** Department of Gastroenterology and Hepatology, Hokkaido University Hospital, North 14, West 5, Kita-ku, Sapporo 060-8648, Japan; kkawakubo-gi@med.hokudai.ac.jp (K.K.); sakamoto@med.hokudai.ac.jp (N.S.)

**Keywords:** endoscopic ultrasound, fine-needle aspiration, biopsy, genome, mutation, biliary tract cancer

## Abstract

The undesired prognosis of biliary tract cancer is mainly attributed to the difficult detection of cancer lesions, including intraepithelial neoplasia and no standard examination for screening. In addition, pathological diagnosis of biliary stricture, whether it is malignant or benign, is not so easy, because of difficult optimal sampling by forceps biopsy and brush cytology, although various devices and methods for pathological diagnosis have been reported. Furthermore, we have to be careful about post-endoscopic retrograde cholangiography pancreatitis when we approach the biliary tract lesion via a transpapillary route. In order to improve the diagnostic accuracy, there have been several studies that indicate the feasibility and efficacy of genomic analysis for accurate diagnosis of biliary tract cancer by using pathological specimens, including endoscopic ultrasound-guided fine-needle aspiration/biopsy (EUS-FNA/FNB) samples. For efficient and precision medicine for patients with biliary tract cancer, future diagnosis and treatment should also be based on molecular and genetic analyses. In this article, we review and summarize the past knowledge and cutting edge of genomic testing for biliary tract cancer, using EUS-FNA/FNB specimens, and indicate some ingenuities in sample processing to promote effective clinical practice and future perspectives.

## 1. Introduction

The undesired prognoses of biliary tract cancers (BTCs) comprising cholangiocarcinoma and gallbladder cancer—5-year relative survival rates of 24.5–28.9% for them in 2021 in Japan [1]—should be improved by the early and accurate diagnosis and efficient treatment of them. However, there are some hurdles to be overcome for their accomplishment. First, it is difficult to diagnose biliary tract cancer early and accurately, because of its difficult approach location for observation and narrow space for biopsy by an endoscope. Especially, the transpapillary approach under endoscopic retrograde cholangiopancreatography (ERCP) also accompanies post-ERCP pancreatitis, ranging from 5% to 10%, which can lead to lethal complications, cholangitis ranging from 0.5 to 3.0%, and cholecystitis ranging from 0.5 to 5.2% in the occurrence rate and so on [2,3]. For the diagnostic abilities of forceps biopsy with/without cytology for malignant biliary stricture under ERCP-guidance, biopsy alone has been reported to have sensitivities (SNs) of 40–60% and specificities (SPs) of 97–100% [4,5,6,7,8,9], while biopsy with cytology has been reported to have SNs of 47–86% and SPs of almost 100% [10,11]. The low sensitivities are problems of biliary forceps biopsy with/without cytology. Second, there has still been no effective treatment other than radical surgical resection with/without adjuvant chemotherapy. The current standard chemotherapies for unresectable BTC have not fully achieved desirable outcomes, with median survival times of 11.7 and 13.5 months [12,13]. The recent report regarding adjuvant chemotherapy for biliary tract cancer with S-1 (an oral 5-fluorouracil prodrug that consists of tegafur, gimeracil (a potent dihydropyrimidine dehydrogenase inhibitor) and oteracil (an inhibitor of phosphorylation of 5- fluorouracil in the gastrointestinal tract) in a 1:0.4:1 molar concentration ratio for 1 year indicated that overall survival (OS) and disease-free survival rates (DFSs) at 1/2 years were 91.2/80.0% and 84.3/77.2%, respectively; however, this single-arm cohort included more than 40% of patients with positive lymph nodes [14]. Meanwhile, any of three prospective randomized clinical trials (RCTs) exploring experimental adjuvant chemotherapy arms (gemcitabine, gemcitabine with oxaliplatin or capecitabine) in patients with resected BTC could not yield superiority of adjuvant chemotherapy in OSs and DFSs, as compared with no adjuvant therapy [15,16,17] Therefore, a future RCT with S-1 should be warranted for validation of the beneficial effect. For efficient and precision medicine for patients with both resectable and unresectable BTCs, future diagnosis and treatment should also be based on molecular and genetic analyses with biopsy, cytology, and surgical specimens. One of the less invasive and complementary methods to obtain materials of biliary lesions is endoscopic ultrasound-guided fine-needle aspiration/biopsy (EUS-FNA/FNB). However, all patients with a biliary lesion are not candidates for EUS-FNA/FNB, because the target is frequently small, non-massive, and located on the route via the bile duct/gallbladder lumen. Meanwhile, in appropriately selected patients with a biliary lesion, EUS-FNA/FNB is feasible and very available, without severe adverse events.

In this article, we review and summarize the cutting edge of molecular and genetic analyses with EUS-FNA/FNB specimens and indicate future perspectives.

## 2. Bile Duct

There have been 20 or more reports regarding EUS-FNA/FNB to pathologically diagnose malignant bile duct lesions, including the intrahepatic and extrahepatic cholangiocarcinoma, which indicated the various SNs (46–100%) and high SPs (92–100%) of malignancy [18,19,20,21,22,23,24,25,26,27,28,29,30,31,32,33,34,35,36,37,38]. Sadeghi et al. conducted a meta-analysis regarding EUS-FNA for malignant biliary stricture, which revealed the pooled SN of 80% (95% confidence interval (CI), 74–86%) and pooled SP of 97% (95% CI, 94–99%), respectively [38]. The total adverse event rate was 1.0% (4/383 cases; 95% CI, 0.29–2.65%) in their study: three of them were mild and included self-controlled bleeding, and one was severe adverse event (biliary peritonitis and procedure-related death). For the additional beneficial effect of EUS-FNA/FNB to ERCP-guided pathological diagnosis, de Moura et al. found in a meta-analysis that the SN of ERCP increased from 58% to 86%; however, the SP of ERCP remained unchanged at 98% [39]. Thus, the lower SN of its pathological analysis should be improved by a novel approach, including molecular and genetic analyses, while careful candidate selection for EUS-FNA/FNB in the biliary lesion is indispensable, as described in the abovementioned death case.

Whole-genome sequencing studies in biliary tract cancer have revealed genomic alterations in some oncogenes and tumor suppressor genes, mainly such as *KRAS*, *TP53*, *CDKN2A* and *SMAD4* [40,41,42]. Nakamura et al. revealed that some genetic mutations in biliary tract cancer can commonly occur, and others can respectively occur according to the cancer site, such as the intrahepatic/extrahepatic bile duct and gallbladder with comprehensive genetic analysis including analysis of whole-transcriptome sequence [40]. According to their report, for common mutations in the biliary tract cancer, including gallbladder cancer, *TP53*, *BRCA1*, *BRCA2*, and *PIK3CA* were detected; for intrahepatic and extrahepatic duct-shared mutations, *KRAS*, *SMAD4*, *ARID1A*, and *GNAS* were detected. For intrahepatic duct-specific mutations, *FGFR2* fusion and *IDH1/2*, *EPHA2*, and *BAP1* mutations were detected; and for extrahepatic duct-specific mutations, *PRKACA*/*PRKACB* fusion, *ELF3*, and *ARID1B* mutations were detected. Some reports [40,41,42,43,44,45,46] and a review by Jain et al. [47] regarding the molecular profiles of bile duct cancer indicated frequencies of some major gene mutations: *TP53*, 44–28% in Asians and 6% in Caucasians; *KRAS*, 12–17%; *SMAD4*, 6–20%; *CDKN2A*, 4–5%; *BRCA1/2*, 3–5%; and *PIK3CA*, 3–5%. Referring to them, we should utilize EUS-FNA/FNB specimens for genetic/molecular diagnosis.

Singhi et al. used biliary brushing and biopsy specimens obtained under ERCP guidance in patients with bile duct stricture [48]. They indicated that targeted next-generation sequencing assay for 28 genes improved the sensitivity of pathological evaluation (48% to 83%) for both biliary brushing and biliary biopsy specimens and detected high-grade biliary dysplasia/malignancy, which cannot be assessed by EUS-FNA/FNB, because of the size/site. Biliary biopsy specimens obtained by ERCP are larger and can harbor more DNA of biliary lesions than those by EUS-FNA/FNB. Therefore, the ERCP-guided (inside) and EUS-guided (outside) approach should complementarily be utilized while considering the approach risk and lesion site.

**Figure 1:**
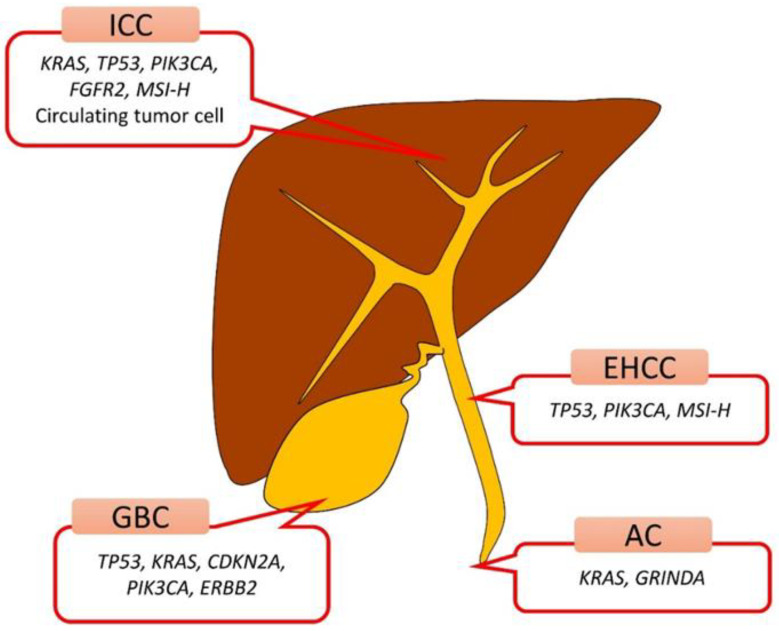
Major genetic mutation spectrum according to the biliary tract site revealed by EUS-FNA/FNB specimens. AC, ampullary cancer; EHCC, extrahepatic cholangiocarcinoma; GBC, gallbladder cancer; ICC, intrahepatic cholangiocarcinoma; MSI-H, microsatellite instability-high.

There have been six reports which revealed the diagnostic accuracy of genetic analysis with EUS-FNA/FNB specimens for biliary tract cancers, including ampullary cancer, extrahepatic/intrahepatic cholangiocarcinoma, and gallbladder cancer (Table 1). The study by Malhotra et al. analyzed *KRAS* oncogene alterations and loss of heterozygosity (LOH) at 10 genomic loci linked to tumor suppressor genes associated with pancreaticobiliary cancer, namely 1p (*CMM1*, *Lmyc*), 3p (*VHL*, *OGG1*), 5q (*MCC*, *APC*), 9p (*CDKN2A*, *CDKN2B*), 10q *(PTEN*, *MXI1*), 17p (*TP53*), 17q (*NME1*, *RNF34*), 18q (*DCC*), 21q (*TFF1*, *PSEN2*), and 22q (*NF2*) in 23 EUS-FNA, and seven biliary brushing specimens from patients with pancreaticobiliary masses [49]. In their analysis, of the nine atypical cytology cases, mutational profiling correctly diagnosed seven cases as positive and one case as negative for aggressive disease (SN in them, 8/9 = 89%), while the total SN and negative predictive value of mutational profiling were low (8/17 = 47%; 9/18 = 50%), and this would be attributed to the site-specific mutational heterogeneity, as Nakamura et al. revealed and sampling limitations. Gleeson et al. analyzed 29 tumors comprising four ampullary adenocarcinoma (AA) and 25 pancreatic cancer with a 160 cancer gene panel and revealed the mutation rates of *KRAS* (93%), *TP53* (72%), *SMAD4* (31%), and *GNAS* (10%); however, the detailed frequencies in AA were not shown [50]. The *SMAD4* mutation was not detected in AA at all. One AA case with stage IIa revealed the *KRAS* and *GRINDA* mutation in an EUS-FNA specimen, while the *KRAS* mutation alone was revealed in a surgical specimen. This discordance may be partially explained by the tumor microenvironment, such as desmoplasia, stroma, or immune cells, and strengthens the significance of genetic analyses with EUS-FNA specimens. Choi et al. analyzed *KRAS* mutation in liver tumors comprising 4 hepatocellular carcinoma, 7 intrahepatic cholangiocarcinoma (ICC), 1 neuroendocrine carcinoma, and 16 metastatic cancer with EUS-FNB specimens and next-generation sequencing [51]. They mainly used 22-gauge core biopsy needles and the “fanning technique” to use the “up/down” endoscope dial or the elevator during the to-and-fro movements and indicated 25% of *KRAS* mutation rate, which was slightly higher than those of the previous results (12–17%); however, it was not sufficient for diagnosis by itself. Meanwhile, Maruki et al. revealed *FGFR2*–FISH (fluorescent in situ hybridization) positive (*FGFR2* rearrangement) rate confirmed by RNA sequencing, 5.4% (23/423; 7.4%, ICC; 3.6%, PCC), in a large Japanese cohort including 272 patients with ICC, 83 patients with perihilar cholangiocarcinoma (PCC), and 68 other BTC patients, using 125 surgical, 177 percutaneous/27 forceps biopsy, and 26 EUS-FNA specimens [52]. The success rate of the FISH assay in the biopsy specimen and surgically resected specimens was 97.5% (273/280) and 100% (152/152), However, Maruki et al. did not show the availability of EUS-FNA specimens in detail. On the whole, a comprehensive gene panel and FISH with appropriately procured specimens could improve the diagnostic ability of EUS-FNA/FNB for BTC and, thus, could lead to precision medicine (Figure 1).

## 3. Gallbladder

There have been only six reports regarding EUS-FNA/FNB to pathologically diagnose gallbladder lesions that firmly verified the utility with the SN and SP [23,53,54,55,56,57,58]. According to the review article including the six reports by Hijioka et al., the SNs and SPs of EUS-FNA for malignancy ranged from 80% to 100% and all 100%, respectively. The adverse event rates in the six reports were all 0%, which is a notable result, although the patient numbers in the included cohorts were mostly small (6 cases in 2 studies; 7 cases in 1 study; 15 cases in 1 study; 83 cases in 1 study; and 101 cases in 1 study). Meanwhile, ERCP-guided approach for gallbladder lesions can be occasionally failed in selective gallbladder cannulation [59] and sufficient pathological sampling, and associated with incidental perforation of the cystic duct in addition to post-ERCP pancreatitis. Therefore, EUS-FNA/FNB for gallbladder lesions can be the first method whenever the puncture via a non-luminal route of the biliary tract can be secured.

According to the report by Nakamura et al. [40], for gallbladder-specific mutations, *EGFR*, *ERBB3*, *PTEN*, *ARID2*, *MLL2*, *MLL3*, and *TERT* mutations were detected. The frequencies of their mutations were reported as follows: *EGFR*, 4–13%; *ERBB3*, 0–12%; *PTEN*, 0–4%; *ARID2*, 3–17%; *MLL2*, 11%; *MLL3*, 11–25%; and *TERT*, 21% [40,47,60]. For other major genes, *TP53*, 32–63%; *KRAS*, 2–59%; *SMAD4*, 6–33%; *BRCA1/2*, 0.3–4%; and *PIK3CA,* 6–13% have been reported [43,60,61,62].

As shown in Table 1, There have been only four reports which revealed the diagnostic accuracy of genetic analysis with EUS-FNA/FNB specimens for gallbladder cancer. We also revealed that pathogenic gene alterations were successfully identified in 20 out of all 21 BTC patients (95.2%) and in 91.7% (11/12) of GBC patients with EUS-FNA/FNB specimens, using 22/25-gauge needles (Lancet needle, namely conventional end-cut needle, for FNA/Franseen needle for FNB) [63]. In GBC samples, *TP53* mutations were most frequent (38%), followed by *KRAS* (21%), *CDKN2A* (9%), *PIK3CA* (4%), and ERBB2 (4%), which were largely compatible with previous reports. We stored 0.5–1.0-mm^3^ portions of the EUS-FNA/FNB white samples, which were considered to include malignant cells near the area of Diff-Quik staining, in RNAlater (Life Technologies, Carlsbad, CA, USA), at 4 °C, immediately after confirmation of malignancy. Namely, it means that our novel rapid on-site process before the library preparation can produce superior sequence data by using targeted amplicon sequencing.

Kai et al. performed a microsatellite instability (MSI) examination by using 15 surgical, 26 percutaneous/5 forceps biopsy specimens, and 12 EUS-FNA specimens of BTC. EUS-FNA was performed for GBC 7, ICC 3, and PCC 2 with 11 Lancet needles (22-gauge) and 1 reverse-bevel needle (20-gauge) [64]. The success rates of MSI examinations were 98.3% (59 of 60) in all specimens and 100% in EUS-FNA specimens, respectively. MSI examination failed in only one case using a surgical specimen due to time-dependent degradation of DNA. However, no MSI-high case was detected in patients with GBC, as the previous reports revealed the low rate of MSI-high cases in gallbladder cancer patients (around 2%) [65,66]. Thus, a comprehensive gene panel with appropriately processed specimens could also improve the diagnostic ability of EUS-FNA/FNB for BTC, which can lead to precision medicine in addition to MSI examination (Figure 1). 

## 4. Needle Size and Sample Volume

According to the review reports by Hijioka et al. [56,58] and Sadeghi et al. [38], almost all lesions in the gallbladder or bile duct pathologically diagnosed by EUS-FNA/FNB were punctured by a 22-gauge or 25-gauge needle. This could be explained by controllability of a needle affected by puncture resistance in the duodenum and small average size of a biliary tract lesion. In the previous reports regarding genomic analysis of EUS-FNA/FNB samples reviewed in this article (Table 1), all lesions, except for one case, were punctured by a 22/25-gauge needle, as far as reported. Therefore, a 22/25-gauge FNA/FNB needle would be feasible and available for the genomic analysis of biliary tract lesions. 

For the tissue amount obtained by an FNA/FNB needle, there have been several comparative studies. For pancreatic lesions (Table 2), Takahashi et al. [67] reported that the 19-gauge Franseen needle obtained significantly more histological tissue samples than the 19-gauge conventional needle (*p* = 0.010) and 22-gauge Franseen needle (*p* = 0.008) (median, [interquartile range: IQR]: 15.20 mm^2^ [6.89–25.75], 5.44 mm^2^ [3.19–25.75], and 4.49 mm^2^ [1.69–6.63]). Conversely, there was no significant difference between the 19-gauge conventional needle and 22-gauge Franseen needle (*p* = 0.838) in this regard. However, all of their target lesions were located at the pancreatic body/tail, because 19-gauge needles are generally difficult to use in the duodenum, as described above. Asokkumar et al. [68] revealed that 22-gauge Franseen EUS-FNB procured significantly more median total tissue area (5.2 mm^2^ vs. 1.9 mm^2^, *p* < 0.001) and diagnostic tissue area (2.2 mm^2^ vs. 0.9 mm^2^, *p* = 0.029) as compared with 22-gauge EUS-FNA in a randomized controlled trial as a single center setting. More interestingly, they indicated that EUS-FNB significantly increased the amount of DNA (median; 2185 [1478–3066] ng vs. 1477 [1151–2522], *p* = 0.08) and RNA (median; 1634 [1089–3939] ng vs. 1295 [986–1782] ng, *p* = 0.02) obtained from the specimen. Mukai et al. [69] also revealed that the presence of desmoplastic fibrosis with neoplastic cellular elements and venous invasion were significantly higher (96.7% vs. 40.0%, *p* < 0.001 and 23.3% vs. 0%, *p* < 0.01, respectively), and the amount of obtained tissue was significantly larger with the 22-gauge Franseen needle as compared with a 22-gauge FNA needle (2.13 mm^2^ vs. 0.45 mm^2^, *p* < 0.001). Bang et al. [70] compared the two types of 22-gauge FNB needles, the Franseen needle and Fork-tip needle, in pancreatic solid masses and indicated that there was no significant difference in the area of total tissue (median 6.1 [IQR, 3.5–10.5] vs. 8.2 mm^2^ [IQR, 4.0–13.0], *p* = 0.50), tumor (median 0.9 [IQR 0.3–2.8] vs. 1.0 mm^2^ [IQR 0.4–2.7], *p* = 0.33), and retained architecture (100% vs. 83%, *p* = 0.25). Namely, the 22-gauge Franseen needle could procure the diagnostic tissue of about 1–4 mm^2^ (approximately 1000–4000 ng/FNB specimen as predicted DNA amount). Although there has been no report on detailed tissue area procured by 25-gauge FNB needle, Oh et al. [71] revealed that the optimal histologic core procurement rate was 87.1% (61/70) for the 25-guage FNB needle vs. 97.1% (68/70) for the 22-gauge FNB needle (*p* = 0.03).

Meanwhile, the initial gene panel (for example, Ion AmpliSeq™ Cancer Hotspot Panel by Life Technologies, Carlsbad, CA, USA) with around 50 genes required at least >10 ng of DNA input volume (concentration, >2 ng/μL), which accounts for 2000 target cells, and could be accomplished even with a tiny specimen without tissue structure obtained by a 22/25-gauge FNA/FNB needle [63,72]. That is a major advantage of genetic analysis with EUS-FNA/FNB samples compared with immunostaining analysis with a surgical/biopsy specimen, which requires a larger sample with sustained tissue structure. The recent gene panels with hundreds of genes (for example, TargetGxOne™ Amplicon Sequencing by Azenta Life Sciences, Chelmsford, MA, USA; FoundationOne^®^ CDx by Foundation Medicine, Inc., Cambridge, MA, USA) under a newest commercial sequencer also require at least >100 ng and 50 ng (>20 ng/μL recommended in TargetGxOne™) of DNA input volume, respectively. Therefore, based on the abovementioned reports on tissue volume, a 22-gauge FNB needle, such as the Franseen or Fork-tip needle, would be appropriate for genetic analysis, although there has been no comparative report on the tissue amount obtained by various sizes of FNB needles.

## 5. Conclusions and Future Perspectives

Many endoscopists have been trying for decades to obtain enough material for the pathological diagnosis of biliary tract cancer, but now it is shifting to molecular/biological diagnosis by tiny specimens. Furthermore, apart from cytology/tissue specimens, Catenacci et al. utilized portal vein blood obtained by EUS-FNA with a 19-gauge needle for the diagnosis of pancreaticobiliary neoplasm via the detection of circulating tumor cells (CTCs) defined as cells positive for cytokeratins 8, 18, and/or 19 and 40,6-diamidino-2-phenylindole [73]. They revealed the CTC detection rate of 100% in 18 patients (14 pancreatic cancer, 1 AC, 1 PCC, 1 DCC, and 1 IPMN), with no adverse event. By using sorted CTCs, tumor genomes could be extracted and utilized for genomic testing, although they did not perform it. Similarly, bile obtained by EUS-FNA might be feasible and available for a novel diagnostic method, because bile should contain much information derived from cancer cells, such as cell-free DNA (cfDNA), micro-RNA, proteome, metabolite, and so on, if the risk of puncture of the bile duct/gallbladder lumen would be overcome. For the genomic analysis of bile, there have been several reports. The study by Shen et al. [74], using targeted deep sequencing with a 150 gene panel, revealed a high SN of 94.7% and an SP of 99.9%. They shed light on a promising bile liquid biopsy with a gene panel for BTC patients. The study by Arechederra et al. [75] also provided us with interesting and informative findings that SN for malignancy of their NGS assay with bile cfDNA was 100% in patients with an initial cytological/pathological diagnosis of benign (gene mutation: *KRAS*, 8/11; *TP53*, 5/11; and *ERBB3*, 4/11 cases) or indeterminate strictures (*KRAS*, 4/7; and *TP53*, 4/7 cases) in addition to a total SN of 96.4% and an SP of 69.2% in their assay, using a 161 gene panel. Namely, a genomic analysis of bile with cfDNA can also harbor information for the detection of early cancer, in addition to a complementary role in the difficult biopsy of cancer. 

Furthermore, new technologies, such as the third- or fourth-generation sequencer, which can detect one molecule of DNA with long-read sequencing, using fixed polymerase in a microwell or nanopore technology [76], and multiplex droplet digital PCR, which can detect multiple gene mutations in tiny specimens [77], have emerged and are beginning to be used on a trial basis. In the near future, these technologies would be feasible in the biliary specimen obtained by EUS-FNA/FNB.

Future validation studies for genomic testing and technical evolutions are expected to result in large and evolutional changes in the detection and diagnosis of early biliary tract cancer. 

## Figures and Tables

**Table 1:** Previous reports on genetic analysis with EUS-FNA/FNB specimens for biliary tract cancers.

Year	Author	Sampling Site	Sampling Method	Patient ** Number	Lesion Size (mm)	Needle Size (Number)	Analysis Target	Analysis Method	
2014	Malhotra et al.	ND	FNA §	ND	ND	ND	LOH of 10 genes and *KRAS* point mutations	PCR and subsequent capillary gel electrophoresis	
2016	Gleeson et al.	Ampulla	FNA ||	4	30 (25–41) ††	ND	160 cancer gene	NGS	
2017	Choi et al.	Liver left lobe	FNB ||	13	33 (23–39) ††	22 G (25); 25 G (3)	*KRAS*	peptide nucleic acid-PCR vs. NGS	
2019	Hirata et al.	BT, LN	FNA/FNB ||	21	ND	22 G (19), 25 G (2)	50 cancer gene	Targeted Amplicon Sequencing	
2021	Maruki et al.	ND	FNA ¶	423	ND	ND	*FGFR2*	FISH + targeted RNA sequencing	
2021	Kai et al.	ND	FNA ¶	60	29 (12–85) ‡‡	22 G (11), 20 G (1)	MSI (BAT-26, NR-21, BAT-25, MONO-27, NR-24)	MSI Kit	
Cancer Type (Number) *		Cancer Stage *		**DNA Extraction**	SN *	**SP**	**ACC**	**PPV**	**NPV**
Pancreatico**biliary mass** (26)		ND		26/26	8/17	9/9	17/26	8/8	9/18
**AC (4)**, PDAC (22), IPMC (3)		Ia 2 (7%) Ib 3 (10%) IIa 4 (14%) IIb 18 (62%) III 1 (4%) IV 1 (3%)		29/47	*KRAS* 93% *TP53* 72% *SMAD4* 31% *GNAS* 10%	NA	NA	NA	NA
HCC (4), **ICC** **(7)**, NEC (1), m-PC (7), m-**GBC (5)**, **m-AC (1)**, m-Others (3)		ND		PCR: 27/28 NGS: 16/28	PCR 14.3% NGS 25%	NA	Pathology + *KRAS* = 96.4%	NA	NA
**p-GBC (10), p-ICC (1), p-EHCC (1); n-GBC (2), n-ICC (5), n-EHCC (2)**		**II/III/IV, 2/2/17**		21/21	**GBC/ICC/EHCC: *TP53*, 38/20/40%; *KRAS* 21/30/0%; *CDKN2A*, 9/0/0%; *PIK3CA*, 4/10/20%; *ERBB2* 4/0/0%**	NA	NA	NA	NA
**ICC, PCC, DCC, GBC, AC**		**IV/recurrence**		†	**ICC 7.4%** **PCC 3.6%**	NA	NA	NA	NA
**ICC (24), PCC (12), DCC (4), GBC (16), AC (4)**		ND		**MSI 59/60**	**MSI-H 3.3% ‡**	NA	NA	NA	NA

BT, biliary tract; FISH, fluorescence in situ hybridization; ND, not described; NGS, next-generation sequencing; FNA, Endoscopic ultrasound fine-needle aspiration; FNB, Endoscopic ultrasound fine-needle biopsy; LN, lymph node; LOH, loss of heterogeneity; MSI, microsatellite instability; PCR, polymerase chain reaction. ** Patients with biliary tract cancer. § FNA and brushing cytology. || FNA/FNB alone. ¶ FNA, surgery, percutaneous/transpapillary biopsy were included. †† The median value (interquartile range) was calculated in all tumors, including non-BT cancer. ‡‡ The median value (range) was calculated in 12 BT cancer examined by FNA. AC, ampullary cancer; ACC, accuracy; DCC, distal cholangiocarcinoma; GBC, gallbladder cancer; PCC, perihilar cholangiocarcinoma; PC, pancreatic cancer; PDAC, pancreatic ductal adenocarcinoma; SN, sensitivity; SP, specificity; PPV, positive predictive value; NA, not assessed; NPV, negative predictive value; m-, metastatic; n-, lymph node; p-, primary. * Bold text means biliary tract cancer-limited items. † The success rate of the FISH assay in the biopsy specimen was 97.5% (273/280). ‡ Two surgical specimens in all 60 samples.

**Table 2:** Relationship between needle size and sample volume in FNA/FNB for a pancreatic/peripancreatic mass.

FNA/FNB Needle	19G-FNB	19G-FNA	22G-FNB	22G-FNA	25G-FNB
Pathological tissue area (mm2) (IQR)	15.20 (6.89–25.75)	5.44 (3.19–25.75)	4.49 (1.69–6.63) 2.2 2.13 0.9 (0.3–2.8)–1.0 (0.4–2.7)	0.9 0.45	NA (Optimal histologic core procurement: 87.1%, 25G-FNB vs. 97.1%, 22G-FNB)
DNA amount (ng)			2185 (1478–3066)	1477 (1151–2522)	
DNA amount (ng) (predicted)	15,000	5000	1000–4000	500–1000	NA

FNA/FNB, fine-needle aspiration/fine-needle biopsy; G, gauge; IQR, interquartile range; NA, not assessed.
